# Glutaraldehyde-crosslinked chitosan-polyethylene oxide nanofibers as a potential gastroretentive delivery system of nizatidine for augmented gastroprotective activity

**DOI:** 10.1080/10717544.2021.1971796

**Published:** 2021-09-02

**Authors:** Walaa Ebrahim Abd El Hady, Osama Abd El-Aazeem Soliman, Hassan Mohamed El Sabbagh, Elham Abdelmonem Mohamed

**Affiliations:** Department of Pharmaceutics, Faculty of Pharmacy, Mansoura University, Mansoura, Egypt

**Keywords:** Nizatidine, chitosan, nanofibers, gastroretention, gastroprotective activity

## Abstract

Nizatidine (NIZ), a histamine H_2_-receptor antagonist, is soluble and stable in the stomach, however, it exhibits a short half-life and a rapid clearance. Therefore, chitosan (CS) and polyethylene oxide (PEO) nanofibers (NFs) at different weight ratios were prepared by electrospinning and characterized. The selected uncrosslinked and glutaraldehyde-crosslinked NFs were investigated regarding floating, solid-state characteristics, *in vitro* release, and *in vitro* cytotoxicity. The cytoprotective activity against ethanol-induced gastric injury in rats was evaluated through macroscopical, histopathological, immunohistochemical, and oxidative stress examinations. NFs based on 8:2 CS:PEO exhibited the smallest diameter (119.17 ± 22.05 nm) and the greatest mucoadhesion (22.82 ± 3.21 g/cm^2^), so they were crosslinked with glutaraldehyde. Solid-state characterization indicated polymers interaction, a successful crosslinking, and NIZ dispersion in NFs. Crosslinking maintained swollen mats at pH 1.2 (swelling% = 29.47 ± 3.50% at 24 h), retarded their erosion at pH 6.8 (swelling%= 84.64 ± 4.91% vs. 25.40 ± 0.79% for the uncrosslinked NFs at 24 h), augmented the floating up to 24 h vs. 10 min for the uncrosslinked NFs at pH 1.2 and prolonged the drug release (%drug released ≥ 93% at 24 h vs. 4 and 5 h for the uncrosslinked NFs at pHs 1.2 and 6.8, respectively). The viability of Caco-2 cells ≥ 86.87 ± 6.86% revealed NFs biocompatibility and unreacted glutaraldehyde removal. Crosslinking of 8:2 CS:PEO NFs potentiated the antiulcer activity (38.98 vs. 8.67 for the uncrosslinked NFs) as well as it preserved the gastric wall architecture, COX-2 expression, and oxidative stress markers levels of the normal rats.

## Introduction

Gastroretentive drug delivery systems (GRDDSs) have been employed to improve the oral delivery of the drugs that showed a solubility in the acidic environment, a high absorption in the upper gut, a degradation in the colon, and a short half-life (Sunoqrot et al., 2017). They offered less fluctuation in the drug blood levels and improved patient compliance (Malik et al., 2015). As well, they reduced the dosing frequency, the side effects, and the overall health care cost. Nanofibers (NFs) have recently emerged as a potential GRDDS since they possess a high surface per unit mass that results in a substantial density reduction required for the floatability. The high surface area is accompanied by an increase in water adsorption and drug solubility. The high charge to volume ratio augments NFs mucoadhesive properties. Also, no stabilization is required making NFs preparation easy to scale up. The nanometric diameter can ensure the adhesion to the mucus layer in the stomach and the high uptake by the macrophages in the inflammation area permitting more prolonged residence at the inflamed tissue, a reduced dose, and a cost-effectiveness as well as an effective gastroprotection against ulceration (Abd El Hady et al., 2019). Also, they are solid dosage forms, so they offer a high stability as well as easy processing, packaging, and shipping (Yu et al., 2010). Nanosized formulations have improved the delivery of various drugs following their ocular (Ameeduzzafar et al., 2014), oral (Zafar, 2020), transdermal (Ahmed et al., 2016; Qumbar et al., 2017), and topical (Ameeduzzafar et al., 2021) uses.

Chitosan (CS) is a naturally occurring linear amino polysaccharide that has been utilized for the drugs encapsulation in nanometric delivery systems because of its biocompatibility, non-toxicity, and permeability-enhancing properties (Abd El Hady et al., 2019). Its mucoadhesive properties can enable a successful gastroretention. Electrospinning is the least expensive and the simplest technique to fabricate NFs. Electrospinning of pure CS is limited possibly due to the formation of strong hydrogen bonds and the hindered free movement of the polymeric chains exposed to the electrical field, and hence a jet break up occurs (Pakravan et al., 2011). The repulsion between its ionic groups is expected to hinder the chain entanglement required for a continuous NFs formation during the jet stretching, bending, and whipping, thus nanobeads are mostly produced instead of NFs (Pakravan et al., 2011). CS electrospinning is also hindered by the high viscosity of its aqueous solution (Bhattarai et al., 2005). Consequently, CS was mixed with an electrospinnable solution of polyethylene oxide (PEO) due to its good biocompatibility and low toxicity as well as its ability to decrease CS viscosity through hydrogen bonding making the solution spinnable (Bhattarai et al., 2005). However, CS electrospun NFs showed a limited mechanical strength in the aqueous media and susceptibility to the enzymatic degradation (Li et al., 2015). Therefore, a chemical crosslinker such as glutaraldehyde was used to bind and couple the functional groups between the polymer chains to preserve the structural integrity *in vivo*, control the drug release rate, and increase NFs tensile strength (Cheng et al., 2015). Glutaraldehyde crosslinks CS NFs through Schiff base formation (Schiffman & Schauer, 2007).

Nizatidine (NIZ) is a histamine H_2_-receptor antagonist used for the treatment of ulcer and gastroesophageal reflux (Jain et al., 2015). It is preferentially absorbed in the parietal cells of the gastric mucosa. NIZ showed a high solubility as well as chemical and enzymatic stabilities in the acidic pH of the stomach, yet its therapeutic efficacy following oral administration is limited due to its short half-life (1–2 h), its metabolism by colonic bacteria and its rapid clearance (Jain et al., 2015). The long-term therapy with H_2_-receptor antagonist and the expected prolonged acid suppression may interfere with the absorption of some drugs, such as lutein, because they require some gastric acid to be absorbed. Hence, the improvement of NIZ delivery and efficacy may reduce the therapy duration and the incidence of this interaction. Accordingly, several studies have been carried out to develop NIZ GRDDSs such as floating pulsatile tablets (Rathnanand & Pannala, 2011), floating osmotic tablet (Sarkar et al., 2014), microballoons (Jain et al., 2015), effervescent gastroretentive tablets (Orugonda et al., 2017), floating tablets (Shahzad et al., 2019), and effervescent floating tablets (Reddy & Tahseen, 2020). These studies did not involve extensive *in vivo* evaluation. Moreover, NFs as a GRDDS offers a lot of advantages over these systems as discussed above. Thus, the purpose of this study was to prepare and investigate the effects of the optimized glutaraldehyde–crosslinked CS:PEO NFs on the delivery and the gastroprotective potential of NIZ against ethanol-induced gastric injury in rats.

## Materials and methods

### Materials

NIZ was kindly supplied by The EL-Nile Co. Pharmaceuticals & Chemical Industries, Cairo, Egypt. PEO with an average molecular weight of 900,000 g/mol, the growth medium RPMI-1640, MTT reagent, and dimethyl sulfoxide was obtained from Sigma-Aldrich, Saint Louis, MO, USA. CS (molecular weight 100,000–300,000 Da) and glycine were purchased from Acros Organics, NJ, USA. Glutaraldehyde aqueous solution (25%) was obtained from Merck, Darmstadt, Germany. All other chemicals were of fine analytical grades. Colorectal adenocarcinoma (Caco-2) cell line was obtained from American Type Culture Collection (ATCC) via holding company for biological products and vaccines (VACSERA), Cairo, Egypt. Fetal Bovine serum was provided by GIBCO, UK. Primary antibodies of COX-2 (Polyclonal PA137504) were purchased from Thermo Fisher Scientific, Waltham, MA, USA. A universal kit of secondary antibodies (0.04% 3, 3′-diaminobenzidine tetrahydrochloride (DAB)) was provided by DAKO, Denmark. Commercial kits for oxidative stress markers were obtained from Biodiagnostic, Egypt.

### NFs preparation

It has been reported that the solutions of low molecular weight CS started to gel above 6% w/v (Desai et al., 2008). In agreement, 5% w/v CS solution in 70% v/v acetic acid was employed to prepare NFs (Mengistu Lemma et al., 2016; Darbasizadeh et al., 2018). In this study, CS (5% w/v) and PEO (2.5% w/v) solutions in 70% v/v acetic acid were mixed to obtain different CS:PEO respective weight ratios of 9:1, 8:2, 7:3, 6:4, and 5:5 (Mengistu Lemma et al., 2016). For example, a volume of 4.5 mL of 5% w/v CS solution equivalent to 225 mg CS was mixed with 1 mL of 2.5% w/v PEO solution containing 25 mg of this polymer to obtain a mixed polymers solution based on 9:1 CS:PEO weight ratio. As a comparison, pure CS solution (5% w/v) was tried to obtain electrospun NFs. The mixed solutions were stirred (Thermolyne Corporation, Dubuque Iowa, USA) at room temperature for 4 h till dissolution. To prepare drug-loaded CS:PEO NFs, accurately weighed NIZ equivalent to 20 mg drug for each mL of the mixed CS:PEO solutions was added and stirred for 2 h, so a drug concentration of 2% w/v was obtained. The prepared mixtures were set aside for 1 h for degassing and then kept in closed bottles at room temperature. NFs were prepared according to a previously adopted method (Bhattarai et al., 2005). The solution was filled into a five-milliliters syringe pump with a needle (gauge 20). The flow rate was 0.3 mL/h and the distance between the collector and the needle tip was 15 cm. A positive voltage range of 25–28 kV was applied. NFs mats were produced on the drum collector that was covered with aluminum foil (NANON-01A, MECC CO., Fukuoka, Japan). NFs mats (5 × 5 cm^2^) were exposed to the vapors of 25% w/v glutaraldehyde solution for 8 h in a desiccator to be then heated at 50 °C under vacuum for 3 h to enhance the crosslinking and remove the remaining glutaraldehyde (Behbood et al., 2018). NFs were washed with 2% glycine aqueous solution and dried at 50 °C under vacuum to remove the unreacted glutaraldehyde (Alhosseini et al., 2012). Finally, the crosslinked NFs were kept in a desiccator.

### Viscosity measurement

The viscosity of the individual and the combined polymers solutions (0.5 mL) was measured at room temperature using a calibrated cone and plate rotary viscometer set at an angular velocity (*N*) of 256 rpm (Haake Inc., Vreden, Germany). Torque value "S (scale grad.)" was determined in triplicate and the results were represented as mean ± SD. The viscosity (*η* in mpa.s) was calculated applying the following equation (Soliman et al., 2017):

$$\eta =\frac{G.S}{N}$$





### Swelling studies

To study NFs swelling profiles, samples were dried and weighed before immersion in test tubes containing 10 mL of 0.1 N HCl (pH 1.2) or phosphate buffer (pH 6.8) at 37 ± 0.5 °C to permit the water uptake (Qasim et al., 2017). At different time intervals of 0.5, 1, 1.5, 2, 3, 4, 5, 6, and 24 h, NFs were removed to be reweighed after the removal of the excess medium on the surface with a filter paper. Swelling (%) was calculated using the weights of the dried (*W*_d_) and the swollen (*W*_w_) NFs mats applying the following equation (Behbood et al., 2018): Swelling (%) = [(*W*_w_–*W*_d_)/*W*_d_] × 100.

### Morphology and size analysis

NFs morphology was observed employing scanning electron microscopy (JSM 6510, JEOL, Tokyo, Japan) with an accelerating voltage of 30 kV. Images were scaled using ImageJ processing software (NIH, USA) to estimate NFs diameter, and their frequency histograms were plotted using Graphpad prism software (Desai et al., 2008). Measurements were done for three SEM images. For each image, the fibers diameter was measured at 25–30 different points.

### Drug content

CS:PEO NFs were evaluated regarding their NIZ content by stirring with 10 mL of 0.1 N HCl for 2 h using 20 mg NFs mat. This was followed by NIZ quantification spectrophotometrically (ultraviolet/visible [UV/VIS] spectrophotometer; JASCO, Tokyo, Japan) at *λ*_max_ of 314 nm against the corresponding plain NFs treated the same to abolish the interference, if any. The experiment was performed in triplicate and the drug content was represented as mean ± SD.

### Mucoadhesive properties

The mucoadhesive properties of NFs mats (2 × 2 cm^2^) at 0.1 N HCl were evaluated using a modified two-arm balance according to a previously adopted method (Desai & Kumar, 2004). Rabbit stomach was collected from a local slaughterhouse. The non-digested food was removed by rinsing with deionized water. The underlying fat and the loose tissues were removed to expose the mucosal layer and the specimens were kept in a physiological saline solution (0.9% w/v NaCl) at 4 °C to be used within 4 h. The preload weight and the preload time were 50 g and 5 min, respectively. The weight of water needed to detach NFs from the mucosa was recorded as the mucoadhesive strength. The experiment was performed in triplicate using a fresh mucosa each time.

### *In vitro* floating study

*In vitro* floating of NFs scaffolds (1 × 1 cm^2^) were placed in a beaker containing 80 mL of 0.1 N HCl (pH 1.2) and kept at 37 ± 0.5 °C. The floating lag time (FLT) was estimated as the time required for NFs to float on the medium surface. The time during which NFs remained floating on the medium surface was recorded and referred to as the total floating time (TFT) (Yusif et al., 2016).

### Solid-state characterization

The tested samples were NIZ, CS, PEO, the selected plain and medicated uncrosslinked NFs as well as the medicated crosslinked NFs in comparison with polymers physical mixtures (PMs) with and without NIZ. Fourier-transform infrared (FT-IR) (Madison Instruments, Middleton, Wisconsin, USA) spectra were traced in the region of 400–4000 cm^−1^. The thermograms were recorded utilizing differential scanning calorimetry (DSC) (model DSC-4, PerkinElmer Inc., Waltham, MA, USA) at 35–400^º^C and a rate of 10 °C/min under a constant dry nitrogen purging at a rate of 20 mL/min. X-ray diffractometry (XRD) was also employed (Diano, Woburn, MA, USA) at 40 kV, 30 mA, and an angle of 2θ.

### *In vitro* drug release study

*In vitro* drug release from 20 mg of the selected uncrosslinked and the crosslinked CS:PEO NFs was investigated in each of 0.1 N HCl (pH 1.2) and a phosphate buffer (pH 6.8) up to 24 h employing the dialysis technique (Mohamed et al., 2018). The actual NIZ content of the examined NFs was determined according to the aforementioned method. Each dissolution medium was stirred at 50 rpm in a thermostatically shaking incubator (Gesellschaft für Labortechnik mbH D-30938 GFL., Burgwedel, Germany) and maintained at 37 ± 0.5 °C. The semipermeable cellophane membrane (12,000–14,000 M. wt. cut off, Fisher Scientific. Co., Pittsburgh, USA) was firstly soaked in the dissolution medium, stretched over the open end of a glass tube having a diameter of 2 cm, and made tight by a rubber band. NFs were placed on the membrane and the tubes were immersed in a 250-mL beaker containing 100 mL of the dissolution medium. Two milliliters were withdrawn at different time intervals of 0.25, 0.5, 1, 1.30, 2, 3, 4, 5, 6, 8, and 24 h and replaced by an equivalent volume of the dissolution medium used. The released drug was determined spectrophotometrically at *λ*_max_ of 314 nm against the corresponding plain NFs treated the same to abolish the interference, if any. Each experiment was carried out in triplicate and the average percentage of drug released was estimated at each time interval.

### *In vitro* cytotoxicity study

*In vitro* cytotoxicity of NFs was evaluated using MTT assay employing Caco-2 cell line (Denizot & Lang, 1986). Cells were cultured in RPMI-1640 medium with 10% fetal bovine serum. Penicillin (100 units/mL) and streptomycin (100 µg/mL) were added. The cell cultures were maintained at 37 ± 0.5 °C in a 5% CO_2_ incubator. The cells were seeded in a 96-well plate at a density of 1 × 10^4^ cells/well and incubated at 37 ± 0.5 °C for 48 h under 5% CO_2_. The cells were then treated with different concentrations (3.13, 6.25, and 12.5 μg/mL) of each of the selected uncrosslinked and crosslinked NFs and incubated for 24 h. Then, a volume of 20 µL of MTT solution (0.5 mg/mL) was added followed by incubation for 4 h. DMSO (100 µL) was added to each well to dissolve the purple formazan formed. The absorbance was measured at 570 nm (*A*_570_) using a microplate reader (ELx 800, BioTek^®^ Instruments, Inc., Vermont, USA). The cell viability% was calculated by dividing the absorbance of the treated samples at 570 nm (*A*_570_) by that of the control then multiplied by 100. Control cells were not exposed to any treatment.

### *In vivo* evaluation

#### Animals

Regulations of US National Institute of Health Guide for the Care and Use of Laboratory Animals reported in NIH publication No. 85–23 and revised in 1996 were followed. Ethical Committee of Faculty of Pharmacy, Mansoura University, Egypt, approved the study protocol. Animals were kept at a temperature of 25 ± 1 °C, under regular 12 h light/12 h dark cycles, and relative humidity of 55 ± 5%. The animals had free access to water and standard laboratory food.

#### Induction of gastric injury and treatment protocol

Thirty rats were randomly divided into five groups. No pretreatment of the rats of groups I (normal control) and II (positive control) was established for 5 d. The other three groups received a pretreatment for successive 5 d with the free NIZ (III) in its aqueous solution at a dose of 30 mg/kg (Adachi et al., 2004), or equivalent doses of the selected NFs either uncrosslinked (IV), or crosslinked (V). All rats had free access to water without food for 24 h on the 5^th^ day. The gastric injury was induced in the rats of groups II, III, IV, and V on the 6^th^ day by a single intragastric instillation of 70% ethanol (10 mL/kg) (El-Maraghy et al., 2015).

#### Tissue collection and preparation

Under deep ether anesthesia, the animals were euthanized 2 h post an intragastric instillation of 70% ethanol. Instant laparotomy was followed by the stomach separation. Each stomach was opened along the greater curvature and rinsed with the normal saline to remove the gastric content and the blood clots. After the examination for any gross gastric injuries, each stomach was divided into two parts. The first part was kept in a buffered formalin solution (10% v/v) for the histopathological examination and the immunohistochemical localization of COX-2. While the second portion was stored at −80 °C and homogenized in 10 volumes of an ice-cold phosphate buffer (100 mM, pH 7.4) for the evaluation of the oxidative stress markers namely malondialdehyde (MDA), superoxide dismutase (SOD), and reduced glutathione (GSH).

#### Macroscopic evaluation

Stomachs were blotted dry to be photographed and examined for gross gastric injury. Paul’s index was calculated by multiplying the percentage incidence of animals with ulcers by the average ulcers number and division by 100. Paul’s index of the positive control (II) was divided by that of each of the treatment groups to estimate AA that was indicated if it was two units or more (Mohamed et al., 2018).

#### Histopathological examination

Following tissues rinsing, they were dehydrated by alcohol, cleared by xylene, and embedded in paraffin in a hot air oven (56 °C) for 24 h. Paraffin blocks were cut into 5 μm sections to be stained with hematoxylin-eosin (H&E) (Bancroft & Gamble, 2007) and examined by a light microscope (Leica Microsystems, Wetzlar, Germany). A qualified pathologist unaware of the specimens' identity performed the histopathological examination to avoid any bias. One centimeter segments of the glandular stomach were examined for the mucosal necrosis (score: 0–3), the edema in the submucosa (score: 0–4), and the inflammatory cells (score: 0–3) (Laine & Weinstein, 1988). The non-glandular portion (1 cm) was screened for submucosal edema and congestion (score: 0–3).

#### Immunohistochemical localization of COX-2

The endogenous peroxidase activity was blocked by 3% H_2_O_2_ for 5 min after paraffin removal with graded xylene and the rehydration in ethanol. For the antigen retrieval, samples were treated with 0.01 M sodium citrate buffer (pH 6.0) in a microwave for 5 min. The slides were then washed with a phosphate buffer (pH 7.2) and incubated overnight at 4 °C in a solution of phosphate buffer containing primary antibodies (1:100) against COX-2. After this, each slide was washed three times with a phosphate buffer and incubated with the secondary antibodies (biotinylated antiimmunoglobulin) for 1 h at room temperature. This was followed by washing and antigen–antibody visualization using 3,3′-diaminobenzidine tetrahydrochloride (DAB) for1 min. The sections were washed and counterstained with Mayer’s hematoxylin to be examined under a light microscope (Leica Microsystems, Wetzlar, Germany) and digital images were captured. The sections treated were scored regarding IHC staining intensity ranging from 0 to 3 in the mucosal and the submucosal layers as follows: absent = 0, weak = 1, moderate = 2, and strong = 3 (Fisher et al., 2005). All readings were blindly performed by a qualified pathologist.

#### Evaluation of gastric MDA, GSH and SOD

Gastric tissues (0.5 g each) were ground with the liquid nitrogen in a mortar, treated with 4.5 mL phosphate buffer (pH 7.4), homogenized on ice, and centrifuged at 4 °C (Heraeus, GmbH, Osterode, Germany). Gastric levels of MDA, GSH, and SOD were estimated in the supernatants using the respective kits according to the manufacturer’s instructions.

### Statistical analysis

Statistical analysis was carried out using GraphPad Software Inc., San Diego, CA, version 5.03 applying one-way analysis of variance (ANOVA), followed by Tukey–Kramer multiple comparisons test at the significance level of *P* < .05.

## Results and discussion

### Viscosity measurement

**Figure 1.**
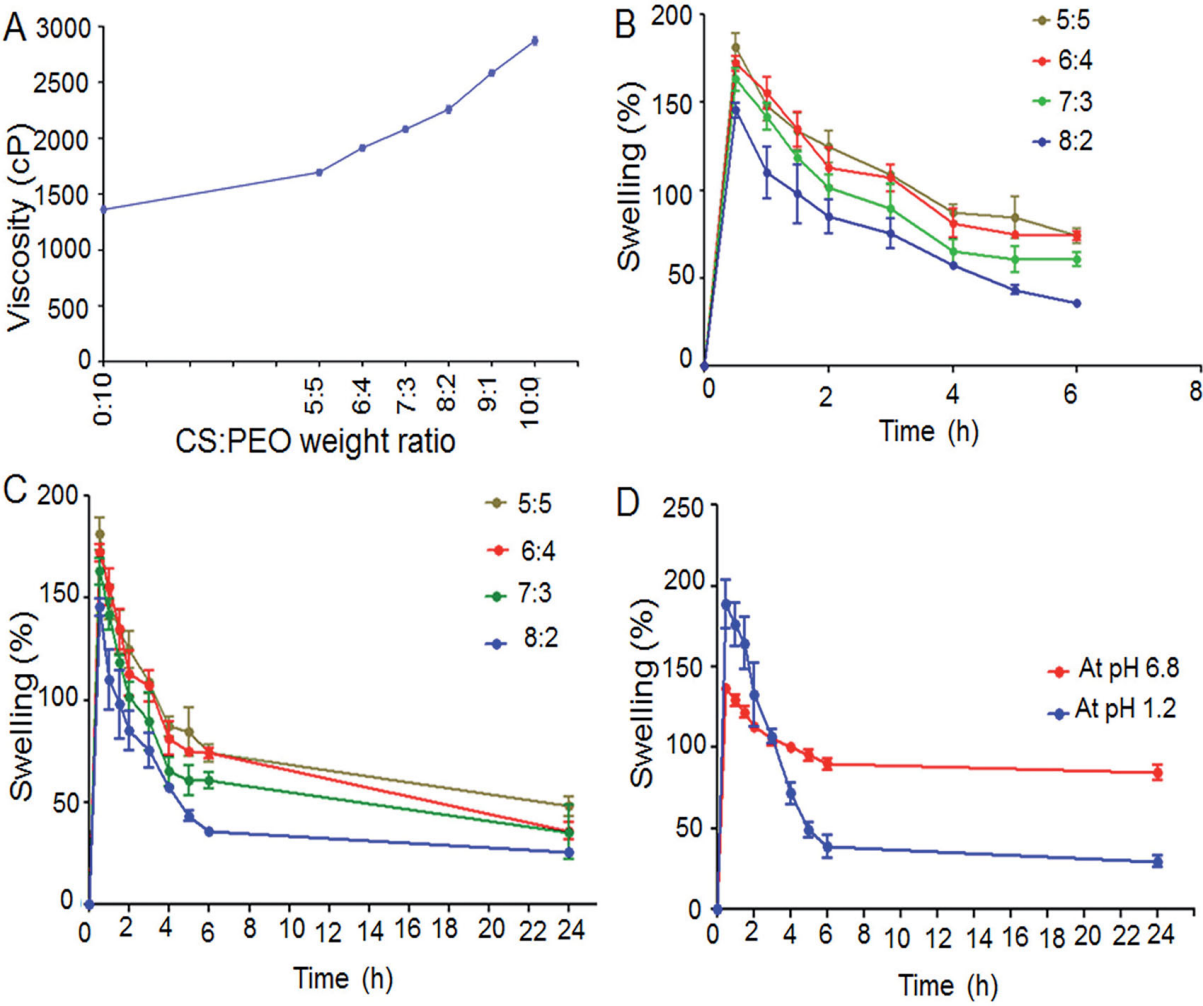
(A) Viscosity of the individual and the mixed polymers solutions using a rotary viscometer at room temperature and an angular velocity of 256 rpm. Swelling profiles of: the uncrosslinked NFs at pH 6.8 up to (B) 6 h, and (C) 24 h as well as (D) glutaraldehyde-crosslinked 8:2 CS:PEO NFs at pHs 1.2 and 6.8 up to 24 h.

The viscosity of solutions containing different weight ratios of CS and PEO was investigated (Figure 1(A)). The high viscosity of the pure CS solution is owing to the strong hydrogen bonding between its OH and NH_2_ groups (Bhattarai et al., 2005). PEO molecules linked to CS backbone could disrupt CS chain self-association and reduce CS solution viscosity (Bhattarai et al., 2005). Small and flexible PEO chains can also lie down along the rigid CS macromolecules smoothing their flow (Pakravan et al., 2011).

### Swelling study

At 0.1 N HCl (pH 1.2), extensive swelling of all NFs occurred quickly followed by a rapid erosion (< 10 min), so swelling (%) was not estimated. The protonation of CS NH_2_ groups at this pH may have disrupted their hydrogen bonding and departed CS chains from each other, and hence, more fluid entered into NFs and a greater swelling was attained (Qasim et al., 2017). The rapid erosion of the swollen NFs can be due to the fast CS degradation at the acidic media and PEO dissolution (Wongsasulak et al., 2014).

Figure 1(B,C) shows swelling (%) of the uncrosslinked NFs at the phosphate buffer (pH 6.8) at 37 ± 0.5 °C for 6 and 24 h, respectively. Swelling% was the maximum at the first 30 min at all CS:PEO ratios. The high water absorption by the hydrophilic polymers of CS and PEO may account for NFs swelling at this nearly neutral medium (Li et al., 2015). The enhanced NFs hydrophobicity due to the poor CS solubility at pH 6.8 could result in a delayed erosion of the swollen NFs (Wongsasulak et al., 2014). However, the accelerated water absorption into the NFs matrix by PEO and its dissolution in the aqueous media would eventually result in a poor perseveration of NFs morphology (Wongsasulak et al., 2014). Therefore, the crosslinking of the selected NFs (8:2 CS:PEO) with gluraldehyde was attempted and the results revealed that it retarded the swollen NFs erosion particularly at pH 1.2 (Figure 1(D)). The aldimine linkages (-CH = N-) between CS free amino groups and glutaraldehyde aldehyde groups can be expected to cause a fusion of the adjacent fibers and retard the water diffusion and NFs erosion (Behbood et al., 2018).

### Morphology and size analysis

**Figure 2.**
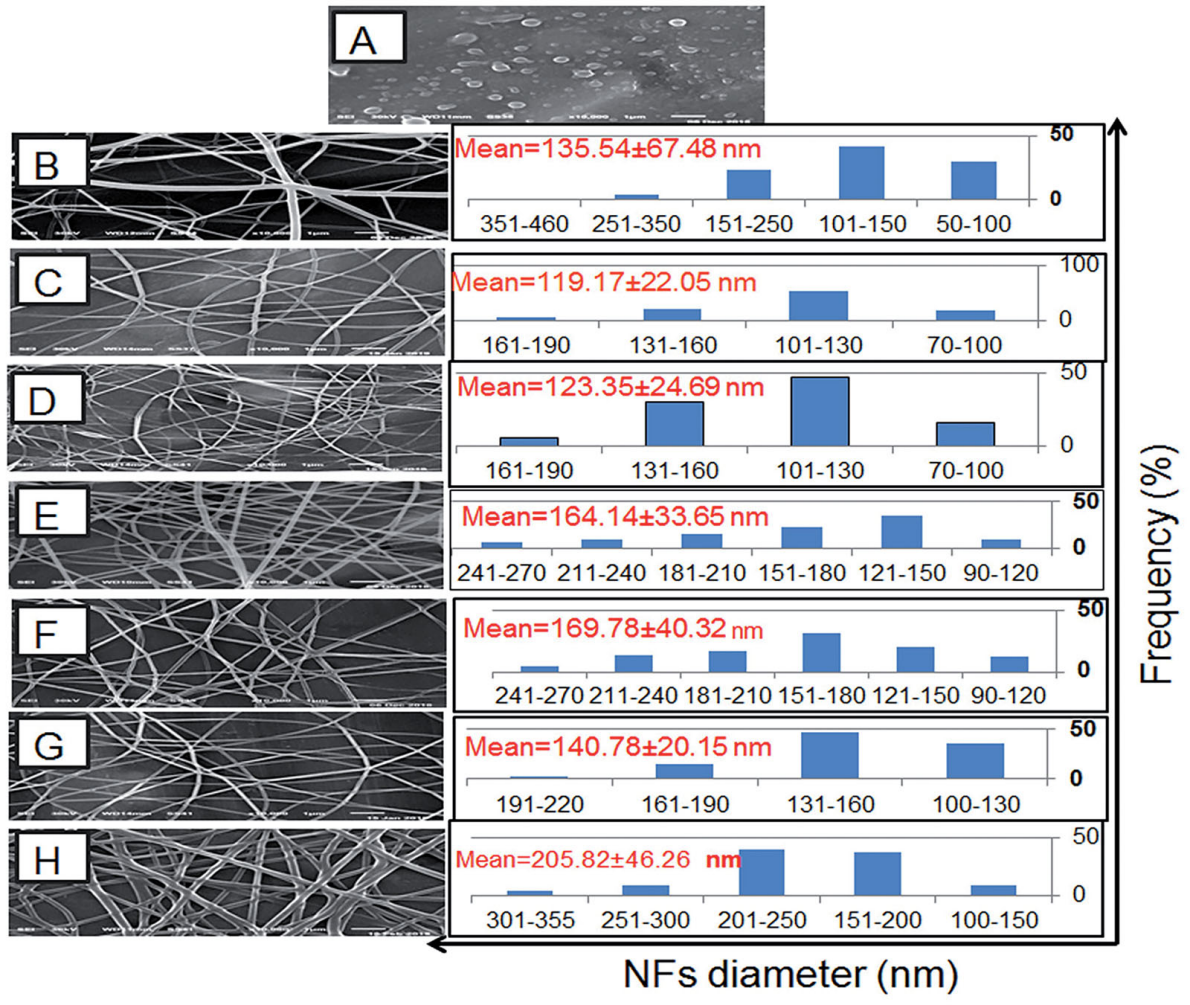
SEM photomicrographs and the corresponding diameter frequency histograms of: (A) CS beads at a concentration of 5% w/v, the uncrosslinked plain NFs based on CS:PEO at respective weight ratios of (B) 9:1, (C) 8:2, (D) 7:3, (E) 6:4, and (F) 5:5. (G) the uncrosslinked NIZ-loaded 8:2 CS:PEO NFs, and (H) glutaraldehyde-crosslinked NIZ-loaded 8:2 CS:PEO NFs.

SEM images of CS:PEO NFs with their diameter distribution histograms are represented in Figure 2. Pure CS solution (5% w/v) formed beads (Figure 2(A)) rather than NFs probably because of its high viscosity that hindered the continuous flow through the syringe tip (Bhattarai et al., 2005). CS mixing with PEO resulted in spinnable solutions and NFs formation (Figure 2(B–H)) possibly due to the hydrogen bonds between PEO polyether oxygen and CS amino hydrogen that lowered the solution viscosity (Pakravan et al., 2011). Accordingly, a spinnable solution containing CS and PEO at a respective weight ratio of 9:1 produced NFs with an average diameter of 135.54 ± 67.48 nm (Figure 2(B)). Compared to 9:1 CS:PEO NFs, the increase in PEO content in 8:2 and 7:3 NFs resulted in significantly smaller diameters (Figure 2(C,D, respectively)) possibly owing to the more decrease in the solution viscosity (Figure 1(A)). Yet, more increase in PEO content in 6:4 and 5:5 CS:PEO NFs resulted in larger diameters (Figure 2(E,F, respectively)). CS can increase the charge density at the ejected jet surface due to the protonation of NH_2_ groups in the acidic medium to NH^+3^ ions resulting in stronger repulsive forces among the polymer chains, and hence a greater elongation force and thinner NFs formation (Alhosseini et al., 2012). Thus, CS content less than a certain limit could diminish its effect on NFs diameter. NFs based on 9:1 CS:PEO were not further investigated due to their largest average diameter and the least size uniformity as reflected by the relatively high SD value. NIZ entrapment into NFs could explain their increased size (Figure 2(G)) (Cui et al., 2018). More NFs enlargement was recorded on crosslinking with glutaraldehyde (Figure 2(H)) possibly due to the linkage with CS and the expected NFs swelling during crosslinking (Cui et al., 2018).

### Drug content

During electrospinning, *in situ* solidification could minimize the drug loss, so the uncrosslinked NFs drug content (%) ranged from 91.46 ± 1.52 to 94.72 ± 1.54% (Table 1). Crosslinked 8:2 CS:PEO NFs showed a significantly lower drug content (87.20 ± 1.47%) than the uncrosslinked ones probably due to the surface drug loss during washing by glycine.

**Table 1.** Drug content and mucoadhesion of NIZ NFs.

CS:PEO weight ratio in NFs	Drug content (%)	Mucoadhesion (g/cm^2)^
5:5	91.46 ± 1.52	10.30 ± 0.71***
6:4	91.73 ± 0.31	15.01 ± 1.55**
7:3	92.03 ± 1.81	17.06 ± 1.12*
8:2	94.72 ± 1.54	22.82 ± 3.21
Glutaraldehyde-crosslinked 8:2	87.20 ± 1.47***	21.36 ± 1.58

Data are expressed as mean ± SD (*n* = 3). ^*^^,**,***^P < .05 indicates a significant difference compared to 8:2 CS:PEO NFs. ^*,**,***^ are the significance degrees *<**<***. NIZ: nizatidine; NFs: nanofibers; CS: chitosan; PEO: polyethylene oxide.

### Mucoadhesive properties

In comparison with the uncrosslinked 8:2 CS:PEO NFs, the forces required to detach the other uncrosslinked NFs from the gastric mucosa were significantly lower (Table 1). The highest CS content in 8:2 CS:PEO NFs could provide more adhesive sites and polymer chains for the interaction with mucin (Patil & Talele, 2015). Therefore, 8:2 CS:PEO NFs were selected to be crosslinked with glutaraldehyde aiming to prevent the fast dissolution in the acidic medium (Pakravan et al., 2011). NFs crosslinking with glutaraldehyde didn’t significantly affect their mucoadhesive properties (Table 1).

### *In vitro* floating study

The uncrosslinked nanofibrous mats floated on the surface of the dissolution medium immediately (FLT < 1 min) and remained floated for about 10 min before degradation at pH 1.2. Meanwhile, glutaraldehyde crosslinked NFs remained floated on the surface of the acidic medium for 24 h following an immediate floating (FLT < 1 min). Therefore, glutaraldehyde-crosslinked 8:2 CS:PEO NFs were further investigated as a GRDDS of NIZ in comparison with the corresponding uncrosslinked NFs.

### Solid-state characterization

#### FT-IR

**Figure 3.**
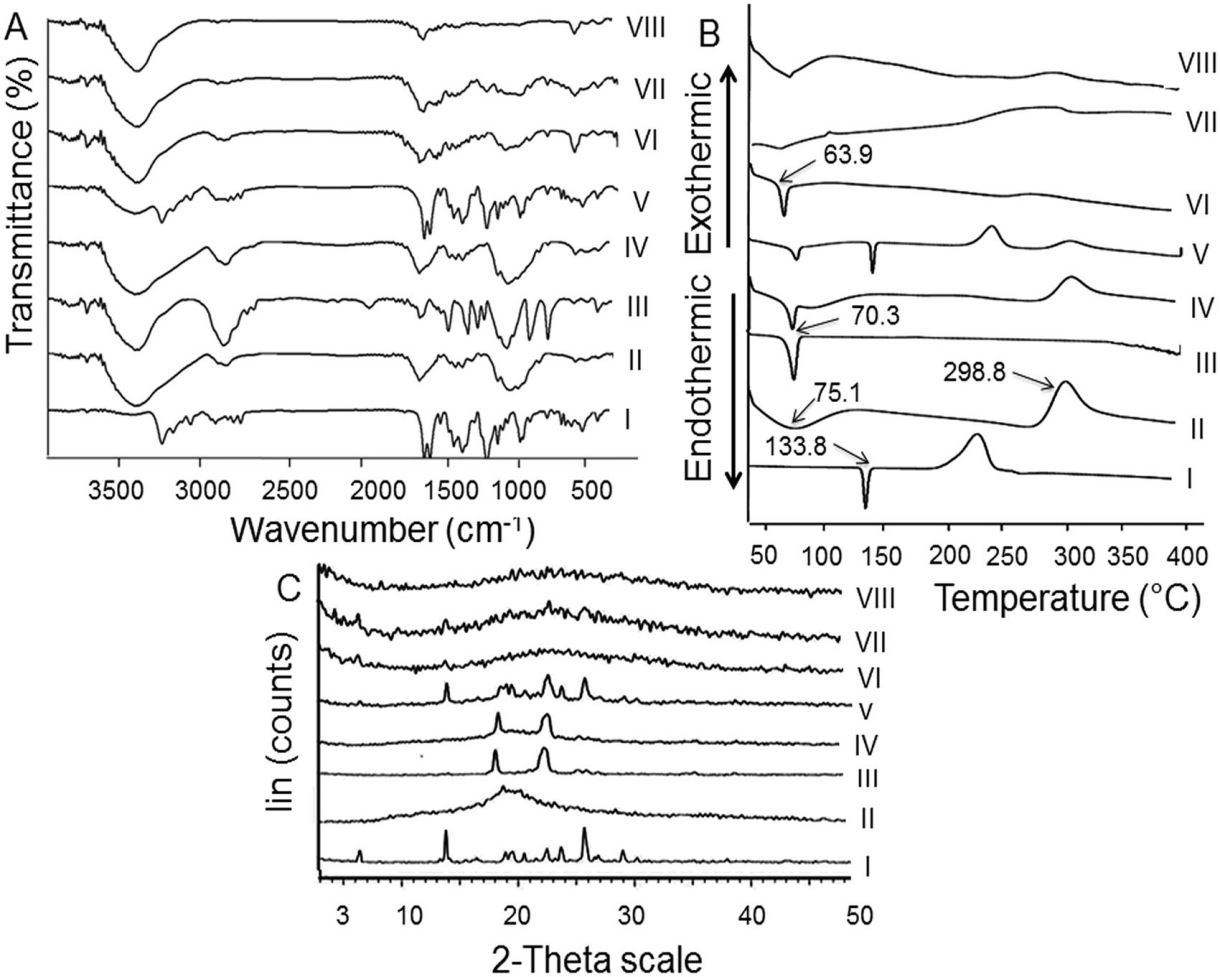
Solid characterization. (A) FT-IR spectra, (B) DSC thermograms, and (C) XRD diffractograms of: (I) NIZ, (II) CS, (III) PEO, (IV) CS:PEO (8:2) PM, (V) CS:PEO:NIZ (8:2:4.8) PM, (VI) the plain uncrosslinked CS:PEO (8:2) NFs, (VII) the uncrosslinked CS:PEO (8:2) NIZ-loaded NFs, and (VIII) glutaraldehyde-crosslinked CS:PEO (8:2) NIZ-loaded NFs.

Figure 3(A) shows FT-IR spectra of the studied samples. NIZ (I) exhibited peaks at 3279, 3098, 1621, 1586^,^ 1520, and 1465 cm^−1^ corresponding to N-H stretch, CH stretch in NO_2_–CH–, C = C conjugated with NO_2_, asymmetrical NO_2_ stretch conjugated with C–C, thiazole ring, and CH deformation in NCH_3_ as well as CH_2_, respectively (Perpétuo et al., 2013). The main characteristic peaks of CS (II) due to N-H and -OH stretching vibrations and intermolecular hydrogen bonding of its backbone were identified at 3447–3550 cm^−1^. The characteristic peaks of CS amide groups (O = CR-NH bending vibration) were detected at 1658 cm^−1^. CH_2_ stretching and CH/OH vibrations were recorded at 2875 and 1424 cm^−1^, respectively. Other peaks at 1157, 1078, and 1030 cm^−1^ were owing to the vibration of C-O-C. These spectral characteristics have been reported (Cheng et al., 2015). The absorption peaks detected at 2889, 1151, 1100, 1059, and 958 cm^−1^ for PEO (III) were attributed to CH_2_ stretching vibration and C-O-C stretching (Cheng et al., 2015).

NIZ peaks were well-detected in the spectrum of its PM with both polymers (IV and V) negating its interaction with any of them following their physical mixing. The spectrum of the medicated uncrosslinked NFs (VII) resembled that of its plain ones (VI) indicating NIZ entrapment. FT-IR analysis verified the successful crosslinking with the glutaraldehyde vapors by identifying a peak of C = N imine at 1629 cm^−1^ in the spectrum describing the crosslinked NFs (VIII) (Schiffman & Schauer, 2007).

#### DSC

DSC thermograms of the investigated samples are illustrated in Figure 3(B). NIZ (I) exhibited a sharp endothermic peak at 133.8 °C which indicates its melting point and its crystalline nature (Bandameedi & Pandiyan, 2016). CS (II) showed endothermic peaks at 75.1 °C and 298.8 °C possibly due to the saccharide rings dehydration and their degradation, respectively (Hanafy et al., 2015). A single sharp endothermic peak appeared at 70.3 °C in PEO thermogram (III) that corresponds to its melting point (Zupančič et al., 2016). The characteristic peaks of PMs components (IV and V) were observed negating their interaction following their physical mixing. The peak of the plain uncrosslinked NFs (VI) at 63.9 °C might refer to their dehydration on heating. This endothermic peak was broadened or shifted to a lower temperature compared to individual polymers owing to the rapid solidification during electrospinning and the expected noncrystalline polymers (Ma et al., 2011). This behavior may also indicate hydrogen bonding between PEO ether and CS amino/hydroxyl groups (Zupančič et al., 2016). NIZ peak disappeared in its NFs thermograms either uncrosslinked (VII) or glutaraldehyde-crosslinked (VIII) indicating NIZ dispersion into them in an amorphous state (Zupančič et al., 2016).

#### XRD

Figure 3(C) depicts XRD patterns of the examined samples. NIZ diffractogram (I) included sharp peaks that reflected its crystallinity (Perpétuo et al., 2013). CS amorphous nature was revealed by the broad peak at 19.58° and the absence of sharp peaks (II) (Agrawal & Pramanik, 2016). PEO exhibited well-defined peaks at 19.08° and 23.2°, indicating its relative crystallinity (III) (Wang et al., 2018). PMs diffractograms (IV and V) exhibited the main diffraction peaks of their components. The diffractograms of CS:PEO NFs after the drug loading (VII) or glutaraldehyde crosslinking (VIII) were similar to the plain NFs (VI) possibly due to the drug dispersion into NFs in an amorphous state (Cheng et al., 2015).

### *In vitro* drug release

**Figure 4.**
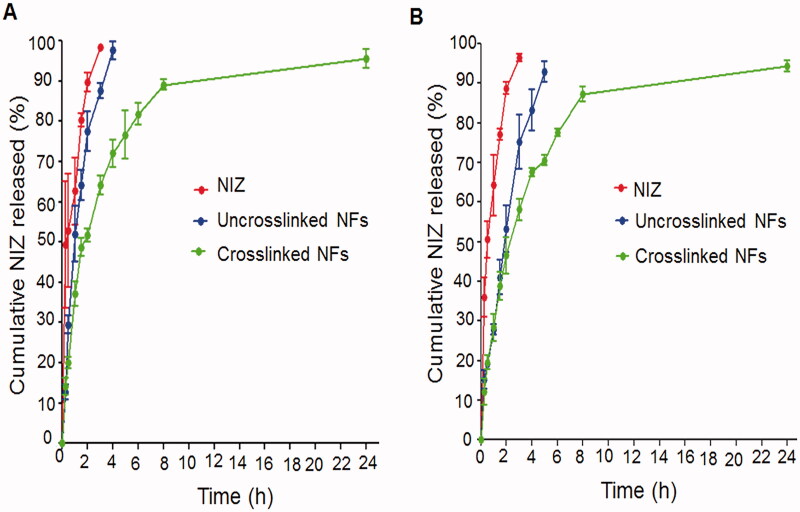
*In vitro* release of NIZ at pHs (A) 1.2 and (B) 6.8. Each point represents the mean ± SD, *n* = 3.

*In vitro* release of NIZ from the selected uncrosslinked and glutaraldehyde-crosslinked CS:PEO NFs was performed at 0.1 N HCl (pH 1.2) and phosphate buffer (pH 6.8) in comparison with the free drug (Figure 4(A,B, respectively)). According to this figure, the free NIZ at both media displayed a burst release (≥ 96.5%) within 3 h possibly due to the fast NIZ dissolution particularly at the acidic medium because of its high hydrophilicity and protonation capability. Similarly, a burst release of NIZ from the selected uncrosslinked NFs could be observed as reflected by an average % drug release of 97.59 ± 2.30% and 83.32 ± 5.19% after 4 h at the media of pHs 1.2 and 6.8, respectively. At pH 1.2, the disruption of CS hydrogen bonding due to the protonation of NH_2_ groups and the subsequent departure of CS chains from each other as well as the increased fluid flow into NFs and the higher swelling rates together can explain the higher drug release at this pH (Figure 4(A)) (Qasim et al., 2017). On the other hand, the enhanced hydrophobicity of CS-based NFs at pH 6.8 would result in a slower swelling and a retarded erosion of the swollen NFs, thus less drug was released (Figure 4(B)).

NFs crosslinking with glutaraldehyde resulted in less drug initially released followed by a gradual release up to 24 h. Only 72.05 ± 3.40% and 67.61 ± 1.03% of NIZ were released from the crosslinked NFs after 4 h at the media of pHs 1.2 and 6.8, respectively (Figure 4(A,B, respectively)). After crosslinking, the linear chain of CS changed to a network chain reducing the swelling and the drug diffusion from these NFs (Behbood et al., 2018). The burst release was also diminished since it was mostly dependent on the medium diffusion into the swollen NFs to release the drug close to the surface. On the other hand, the drug inside the crosslinked NFs had to diffuse first to the surface to be then released, thus it experienced a slower release. The initial burst release can provide the required therapeutic levels of the drug that can be maintained by its subsequent gradual release (Mohamed et al., 2018).

### *In vitro* cytotoxicity study

**Figure 5.**
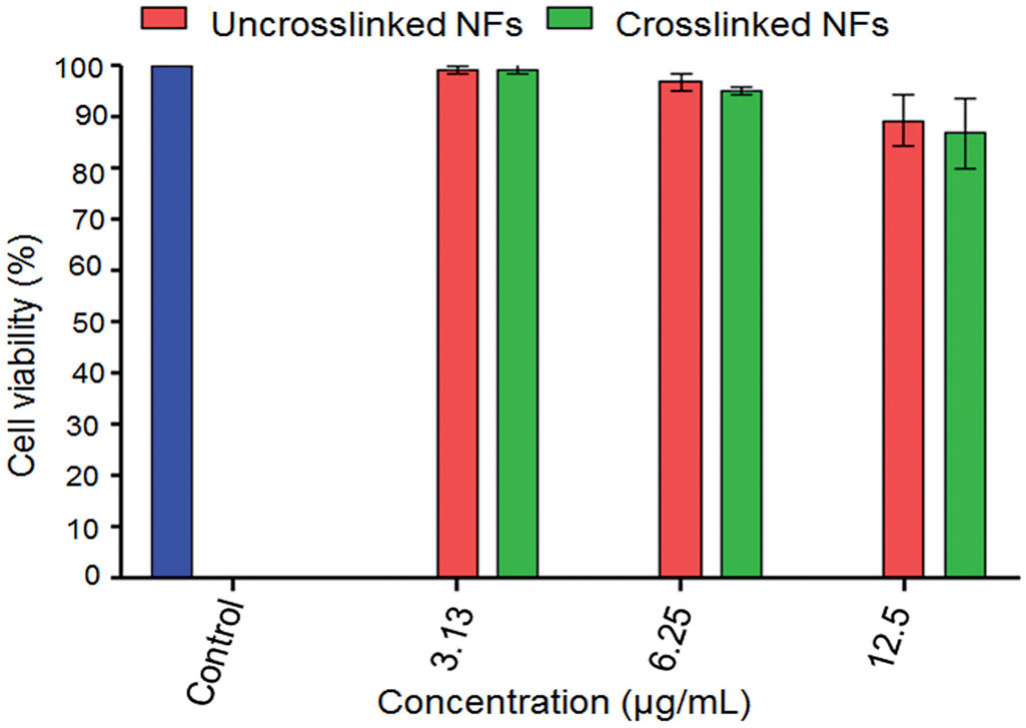
*In vitro* cytotoxicity against Caco-2 cell line using MTT assay. Each point represents the mean ± SD, *n* = 3.

Cell viability ≥ 86.87 ± 6.86% was obtained in the case of the plain NFs either uncrosslinked or glutaraldehyde-crosslinked at the three concentrations used indicating no noticeable toxicity against Caco-2 cells (Figure 5). The viability (%) of Caco-2 cells exposed to both NFs didn’t significantly differ at all concentrations used. This may indicate that CS and PEO are biocompatible and biodegradable as well as washing of NFs with glycine completely removed the unreacted glutaraldehyde.

### Gastroprotective activity evaluation

#### Macroscopic examination

**Figure 6.**
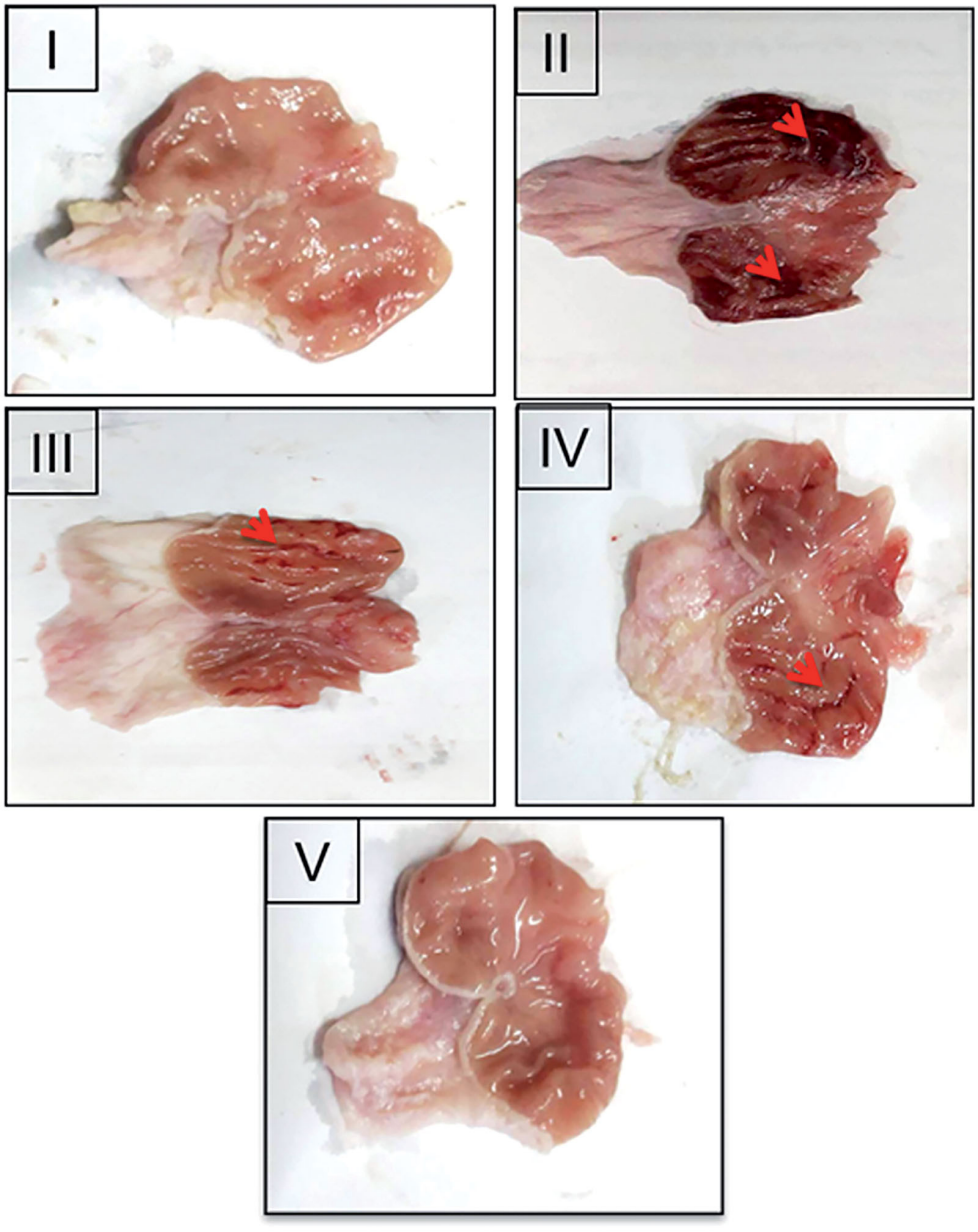
Gross appearance of the freshly excised stomachs of: (I) the normal control, (II) the positive control, the rats pretreated with (III) NIZ (30 mg/kg), an equivalent dose of (IV) the uncrosslinked 8:2 CS:PEO NFs and (V) the crosslinked 8:2 CS:PEO NFs. Red arrows point to the glandular mucosal congestion and ulceration.

Figure 6 represents the gross appearance of the freshly excised gastric tissue of the different groups. The parameters describing AA through macroscopic examination are shown in Table 2. Normal control (I) showed an apparently normal gastric mucosa. Meanwhile, the glandular mucosal ulceration and congestion were extensive in the positive control rats (II). Accordingly, the highest Paul’s index was estimated in the case of the positive control. Rats that were orally given NIZ as a free drug or uncrosslinked NFs (III and IV, respectively) encountered moderate glandular mucosal ulceration, edema, and congestion. Normal gastric mucosa was observed in the rats pretreated with the crosslinked NFs (V). The pretreatment groups (III, IV, and V) experienced a significantly lower mean ulcers number than the positive control (II). Relative to the positive control, highly diminished ulcer incidence and Paul's index values were obtained for these groups. Crosslinking of CS:PEO NFs (V) with glutaraldehyde resulted in a highly potentiated AA of NIZ when compared to the rats pretreated with the free NIZ (III) or the uncrosslinked NFs (IV) probably owing to the more prolonged residence time and the accumulation at the inflamed gastric tissue as well as the enhanced floating, mechanical strength, and mucoadhesion.

**Table 2.** Parameters of macroscopical evaluation of ethanol-induced gastric injury in rats.

Animal group	Number of ulcers Mean ± SD *n* = 6	Percentage incidence of animals	Paul’s index	AA	Severity of inflammation
I (normal control)	–	0	0	–	− (no inflammation)
II (positive control)	26 ± 2	100	26	–	+++ (severe)
III ( NIZ solution)	8 ± 4.81**	50	4	6.5	++ (moderate)
IV (uncrosslinked 8:2 CS:PEO NFs)	6 ± 3.1***	50	3	8.67	++ (moderate)
V (glutaraldehyde-crosslinked 8:2 CS:PEO NFs)	2 ± 1.26***	33.33	0.667	38.98	+ (mild)

30 mg/kg NIZ or an equivalent dose of NFs was used. ^**,***^P < .05 indicates a significant difference compared to positive control. ^**,***^ are the significance degrees **<***. NIZ, nizatidine; NFs: nanofibers; CS: chitosan; PEO: polyethylene oxide; AA: anti-ulcer activity.

#### Histopathological examination

**Figure 7.**
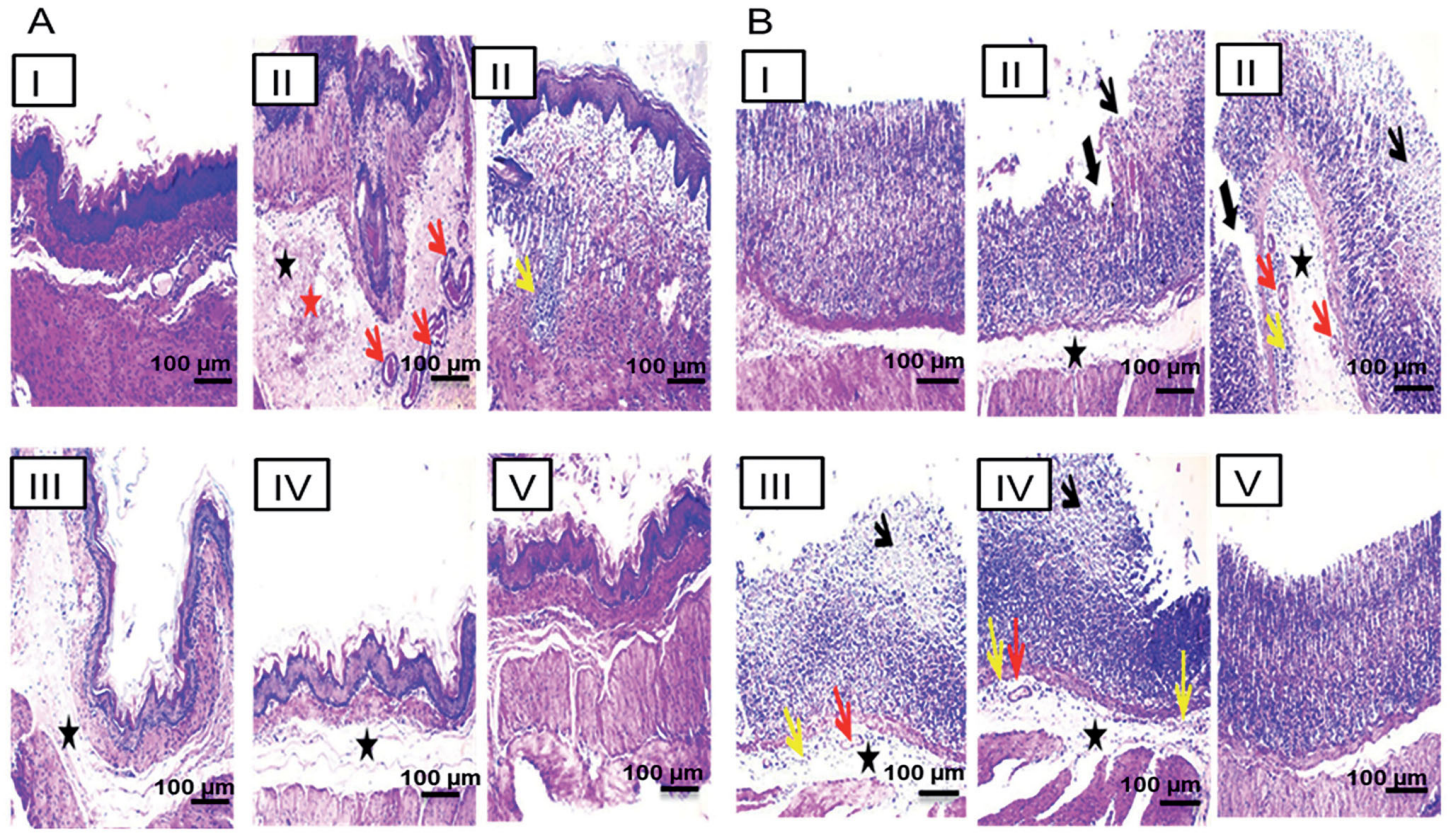
Histopathological examination (hematoxylin and eosin stained, 100x) of the gastric tissue of (A) the non-glandular and (B) the glandular gastric mucosa. I, II, III, IV, and V are as under Figure 6. Submucosal edema (black asterisk), congestion (red arrow), hemorrhage (red asterisk), leukocytic cells infiltration (yellow arrow), mucosal necrosis (thin black arrow), and ulceration (thick black arrow).

Figure 7 displays the photomicrographs of the histopathological examination (HE, 100x) of the non-glandular (A) and the glandular (B) gastric tissues of the different groups. The normal control showed normal non-glandular (IA) and glandular (IB) gastric mucosae. The positive control exhibited marked submucosal edema, congestion, hemorrhage, and leukocytic cells aggregation in the non-glandular portion (IIA) associated with extensive mucosal necrosis, ulceration, submucosal edema, congestion, and leukocytic cells infiltration in the glandular portion (IIB). Oral pretreatment with either the free NIZ or the uncrosslinked NFs caused moderate edema in the non-glandular portion (III and IVA, respectively) in addition to mucosal necrosis with mild submucosal edema, congestion, and inflammatory cells infiltration in the glandular portion (III and IVB, respectively). The non-glandular and the glandular gastric walls retained their normal histological pictures in the rats of group V that orally received the crosslinked CS:PEO NFs (VA and VB, respectively). Accordingly, it can be concluded that the oral pretreatment with NIZ as either a free drug or NFs lowered the pathologic scores and attenuated the gastric damage. Preservation of the non-glandular and the glandular gastric wall architectures was observed only in the rats of group V that were orally pretreated with the crosslinked CS:PEO NFs reflecting their superiority.

**Figure 8.**
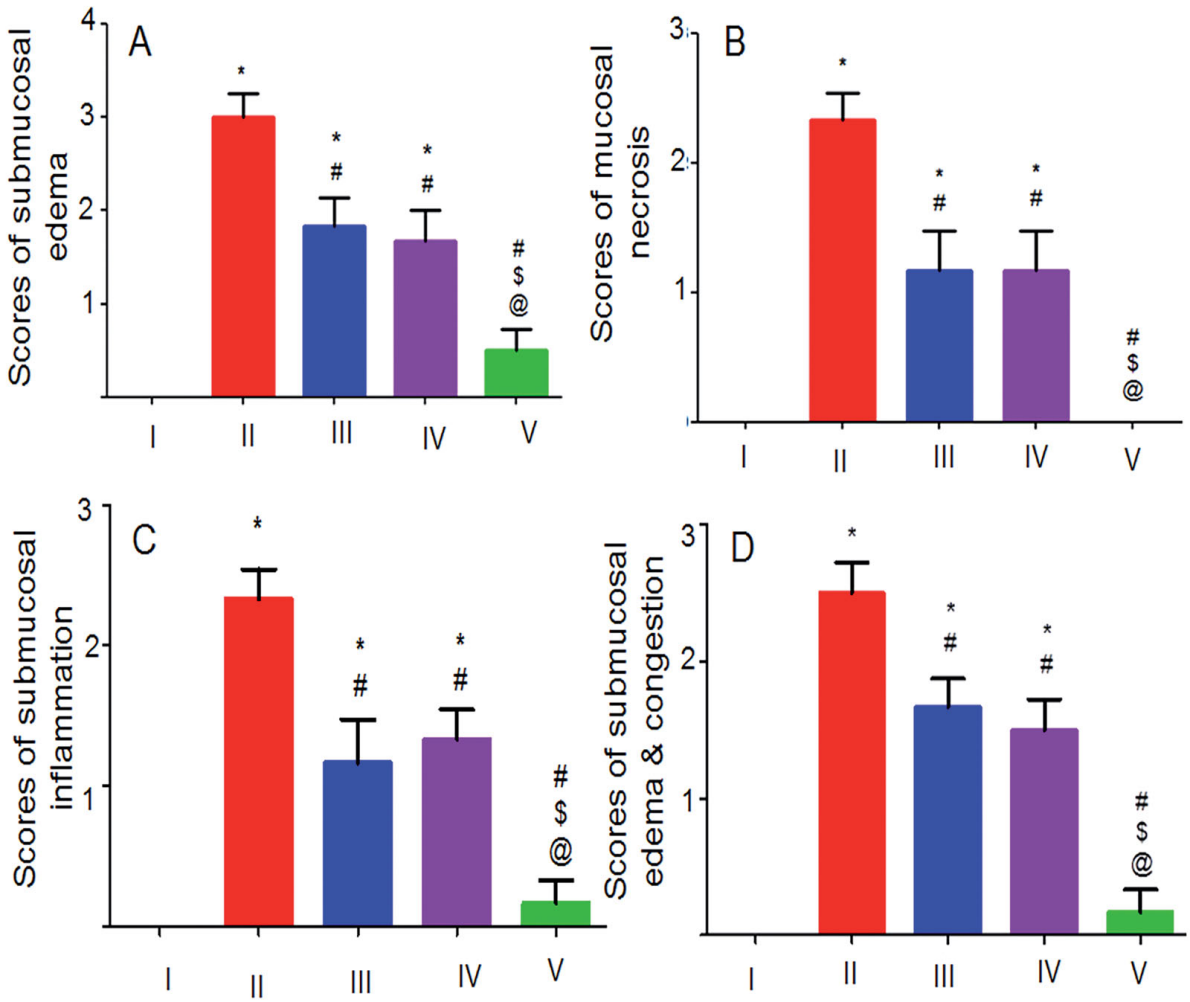
Statistical analysis of the histopathological alterations of (A–C) the glandular mucosa and (D) the non-glandular mucosa. I, II, III, IV, and V are as under Figure 6. Data are mean ± SD, *n* = 6. Statistical differences at *P* < 0.05 were considered significant; *vs the normal control; ^#^vs the positive control; ^$^vs NIZ (30 mg/kg) pretreated rats; ^@^vs the group pretreated with an equivalent dose of the uncrosslinked 8:2 CS:PEO NFs.

The statistical analysis results of the histopathological alterations in the glandular (Figure 8(A–C)) and the non-glandular (Figure 8(D)) gastric tissues were in agreement with the aforementioned findings. There was an insignificant difference between the normal control rats and those that received the crosslinked NFs regarding the severity of all pathological changes describing the ulceration in both the glandular and the non-glandular gastric tissues. Thus, it can be said that the crosslinking of CS:PEO NFs with glutaraldehyde vapors significantly potentiated the cytoprotective activity of NIZ against ethanol-induced gastric injury in rats.

#### Immunohistochemical localization of COX-2

**Figure 9.**
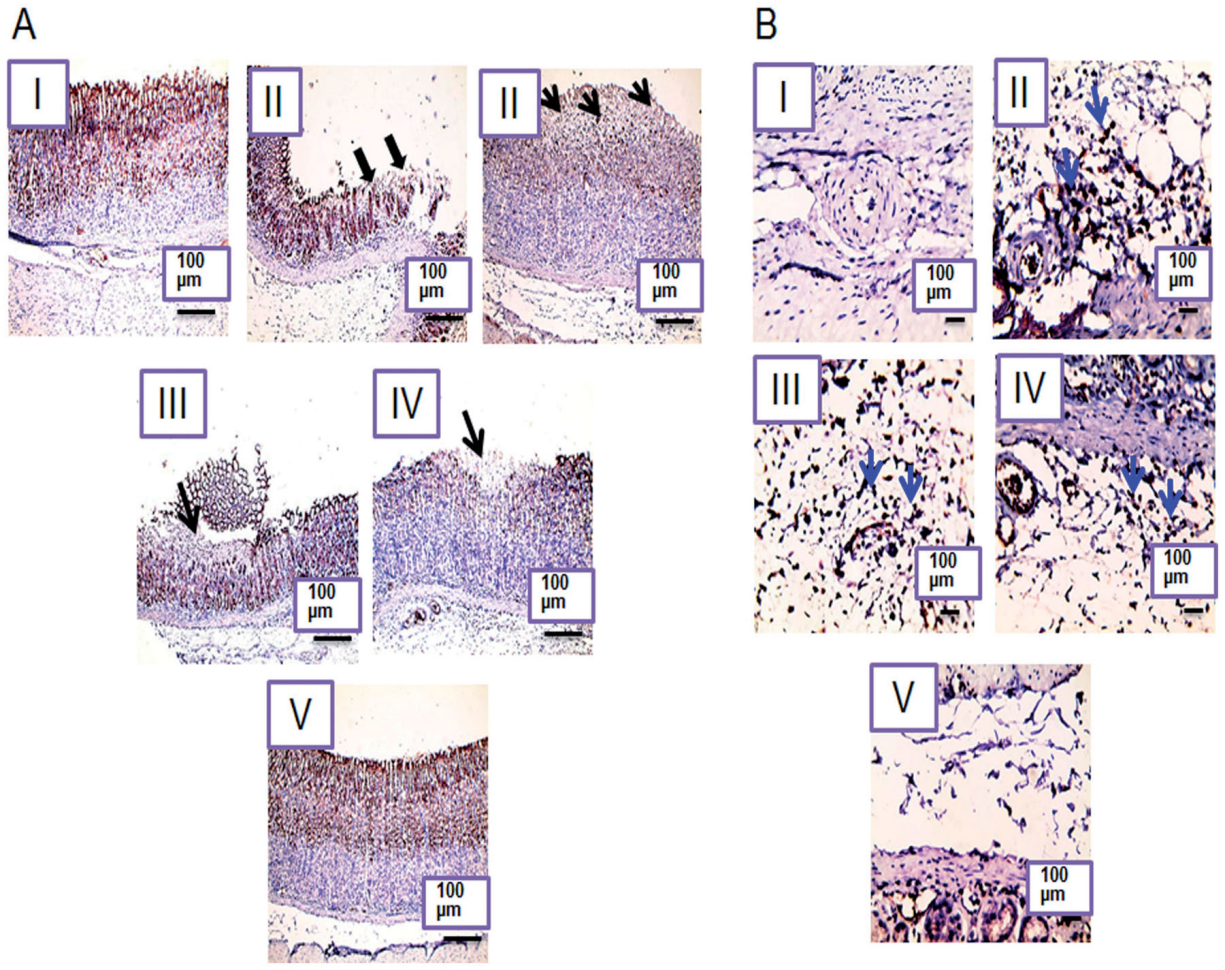
Photomicrographs of rat's stomachs immunostained against COX-2 in (A) the glandular gastric mucosa and (B) the submucosal inflammatory cells. I, II, III, IV, and V are as under Figure 6. X: 100 bar 100 (the glandular gastric mucosa) and X: 400 bar 50 (the submucosal inflammatory cells). Rim of ulcer (thick black arrows), area of mucosal necrosis (thin black arrows), positively stained submucosal infiltrating inflammatory cells (blue arrows).

Immunohistochemical localization of COX-2 was investigated only in the glandular gastric mucosa and submucosa (Figure 9(A,B, respectively)) due to its minimal expression in the non-glandular stomach (Haworth et al., 2005). A positive signal of COX-2 was recognized in the glandular gastric mucosa in the normal control rats (IA) as indicated by the brown staining but no COX-2 expression was recorded in the submucosa (IB) due to its important role in the mucosal defense through prostaglandins production induction (Perini et al., 2003). Prostaglandins counteract gastric injury by inhibiting leukocyte adherence and maintaining the gastric blood flow as well as inducing the angiogenesis and the growth factors (Perini et al., 2003). On the other hand, COX-2 was highly expressed in the submucosa of the positive control rats (IIB) as indicated by the positive staining and the high inflammatory cells infiltration. COX-2 expression is induced under the influence of cytokines and endotoxins at the site of inflammation (Chakraborty et al., 2012). COX-2 expression was less recognized in the mucosal cells at the positive control rats at the ulcer rim and absent in the area of the mucosal necrosis (IIA). The oxidative products of prostaglandins due to ethanol treatment can cause vasoconstriction and platelets aggregation that can aggravate tissue oxidation and the ulcerogenic action of the necrotizing agent (Sametz et al., 2000).

**Figure 10.**
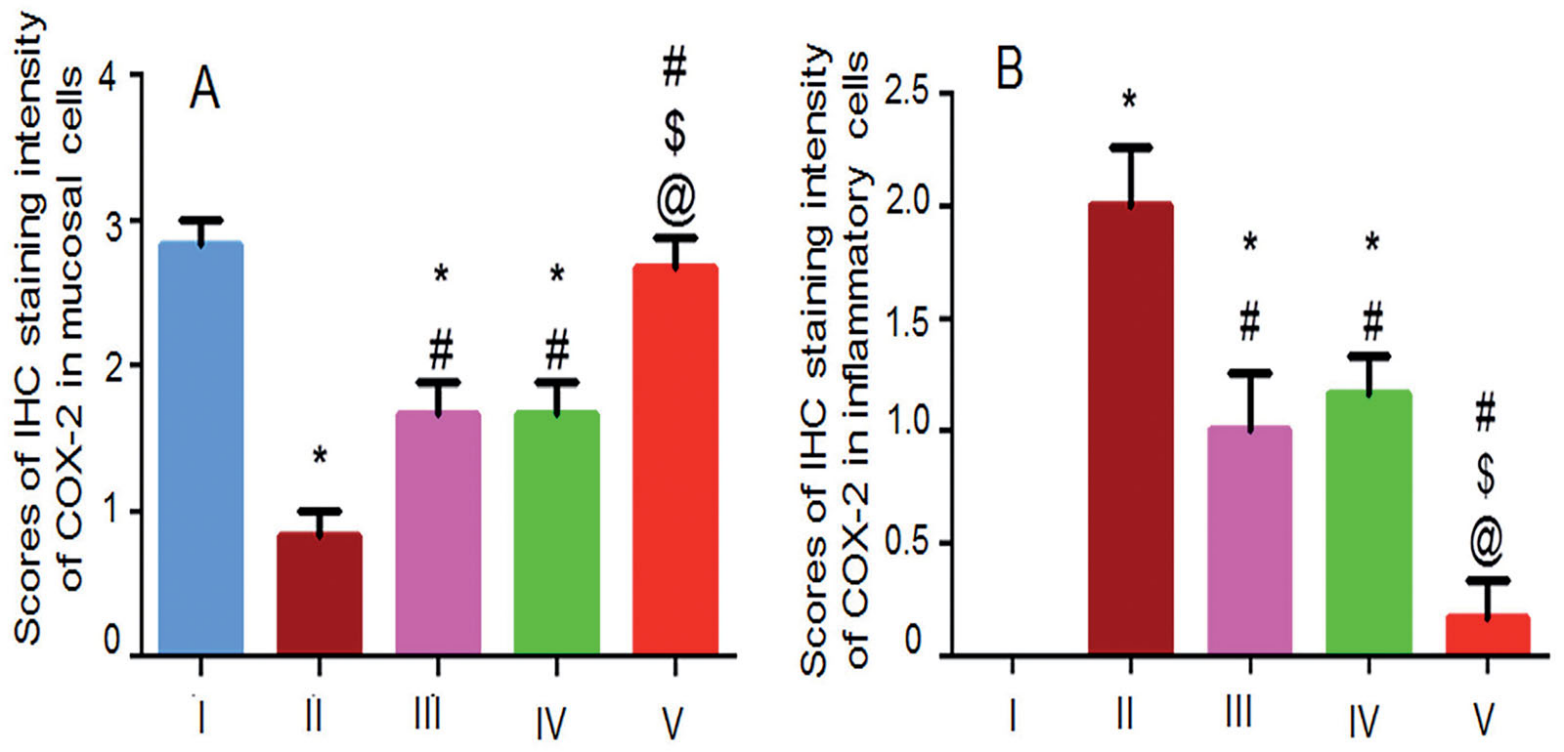
Statistical analysis of IHC staining intensity of COX-2 in (A) the glandular gastric mucosa and (B) the submucosal inflammatory cells. I, II, III, IV, and V are as under Figure 6. Data are illustrated as mean ± SD, *n* = 6. Statistical differences at *P* < 0.05 were considered significant. *, ^#^, ^$^, ^@^ as under Figure 8.

Oral pretreatment with the free NIZ (IIIA) or an equivalent dose of the uncrosslinked NFs (IVA) resulted in a more recorded mucosal expression of COX-2 relative to the positive control rats possibly due to the reduced necrosis as indicated by histopathological examination (Figure 7) and the statistical analysis (Figure 8(B)). Accordingly, less submucosal positive signaling and infiltrating inflammatory cells were observed in these groups (Figure 9(III, IVB, respectively)). Mucosal and submucosal COX-2 expression in the group that received the crosslinked NFs (V) resembled that of the normal control rats (I) confirming the relevant potentiation of the gastroprotective action of NIZ due to the crosslinking of its NFs with glutaraldehyde. The statistical analysis results of the immunohistochemical localization of the gastric COX-2 in the mucosa and the submucosa (Figure 10(A,B, respectively)) were in agreement with the above-mentioned findings. The enhanced floating and the mechanical strength of the selected NFs following their crosslinking may still account for their superiority.

#### Evaluation of gastric MDA, GSH and SOD

**Figure 11.**
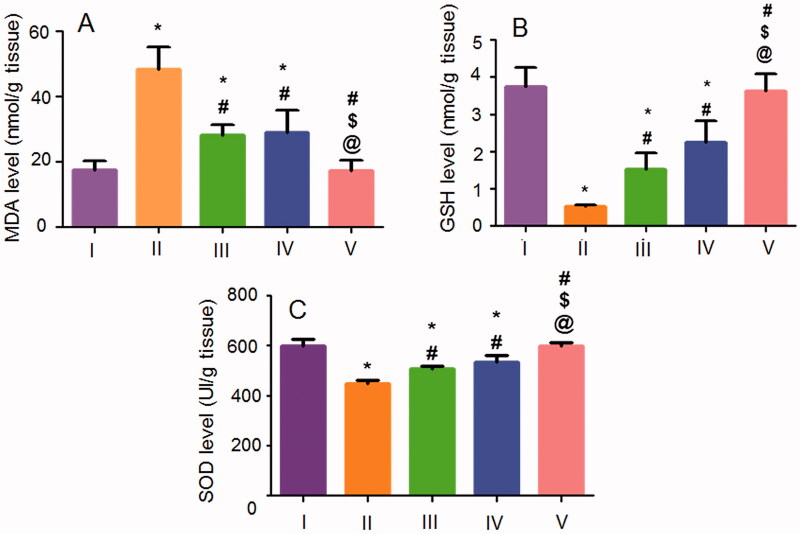
Effects of NIZ oral pretreatment on ethanol-induced changes in oxidative stress markers of (A) MDA, (B) GSH, and (C) SOD in the gastric tissue. I, II, III, IV, and V are as under Figure 6. Data are mean ± SD, *n* = 6. Statistical differences at *P* < 0.05 were considered significant. *, ^#^, ^$^, ^@^ as under Figure 8.

The oxidative stress markers are critically involved in the pathogenesis of gastric ulceration (Mohamed et al., 2018). The gastric levels of the oxidative stress markers including MDA, GSH, and SOD in the studied groups are shown in Figure 11. Ethanol (70%, 10 mL/kg) intragastric administration to the positive control rats induced a significant rise in MDA content (Figure 11(A)) accompanied by a significant reduction in GSH (Figure 11(B)) and SOD (Figure 11(C)) activities when compared to the normal rats. Rats that orally received the free NIZ or the uncrosslinked NFs experienced a significant lowering in MDA content together with a significant elevation in GSH and SOD activities when compared to the positive control rats. The superiority of the crosslinked NFs over the free NIZ and the uncrosslinked NFs was prominently evident by the significant suppression of MDA content and the significant elevation of the antioxidant markers (SOD and GSH) levels being insignificantly different from those shown by the normal rats.

## Conclusions

The use of PEO in the combination with CS at different weight ratios produced well-defined NFs rather than beads. Among the prepared NFs, those based on 8:2 CS:PEO possessed the smallest diameter and the highest mucoadhesion, thus they were chosen to be crosslinked with glutaraldehyde. NIZ molecular dispersion in the selected NFs either uncrosslinked or crosslinked was revealed by FT-IR, DSC, and XRD. The crosslinking delayed the swollen NFs erosion, enhanced their floating, extended the drug release, and potentiated the gastroprotective activity of NIZ as well as maintained the normal gastric wall architecture, COX-2 expression, and the gastric tissue content of the oxidative stress markers. The biocompatibility of NFs components and the successful removal of the unreacted glutaraldehyde by glycine were indicated by the viability (%) of Caco-2 cells that was ≥ 86.87 ± 6.86%. This study may highlight the glutaraldehyde-crosslinked NFs that is based on 8:2 CS:PEO as a potential GRDDS of NIZ and encourage their clinical investigation for a more effective gastroprotective activity of this drug.
